# Crystal structure of *rac*-4-[2-(*tert*-butyl­aza­nium­yl)-1-hy­droxy­eth­yl]-2-(hy­droxy­meth­yl)phenol benzoate

**DOI:** 10.1107/S2056989017011513

**Published:** 2017-08-11

**Authors:** Wenju Liu, Qiliang Chen, Linda Yu

**Affiliations:** a School of Chemical Engineering and Environment, Henan University of Technology, Zhengzhou 450001, People’s Republic of China

**Keywords:** crystal structure, salbutamol benzoate, hydrogen bonds

## Abstract

The title salt, C_13_H_22_NO_3_^+^·C_7_H_5_O_2_^−^, comprises one salbutamol cation {sys­tematic name: 4-[2-(*tert*-butyl­aza­nium­yl)-1-hy­droxy­eth­yl]-2-(hy­droxy­meth­yl)phenol} and a benzoate anion. The cation shows disorder of the hy­droxy group [occupancy ratio 0.738 (3):0.262 (3)] at the stereogenic C atom. The non-planar benzoate anion [the dihedral angle between the benzene ring and the carboxyl group is 11.30 (8)°] is linked to the salbutamol cation by a medium-strength O—H⋯O hydrogen bond. Other inter­molecular O—H⋯O and N—H⋯O hydrogen bonds of weaker nature give rise to [001] chains.

## Abstract 2

The title salt forms a racemate due to disorder of the hy­droxy group [occupancy ratio 0.738 (3):0.262 (3)] at the stereogenic C atom.

## Chemical context   

Salbutamol {systematic name: 4-[2-(*tert*-butyl­amino)-1-hy­droxy­eth­yl]-2-(hy­droxy­meth­yl)phenol} is known as a short-action selective β2-adrenergic receptor agonist for the treatment of pulmonary diseases, including asthma attacks, exercise-induced bronchoconstriction and chronic obstructive pulmonary disease (Saleh *et al.*, 2000 ▸). However, salbutamol shows poor solubility in aqueous solution, which limits its bioavailability. The production of salt forms is a usual approach to alter the physicochemical properties of pharmaceutical compounds (Surov *et al.*, 2015 ▸). Salbutamol has been widely studied and some salts of salbutamol have been on the market, such as salbutamol sulfate.
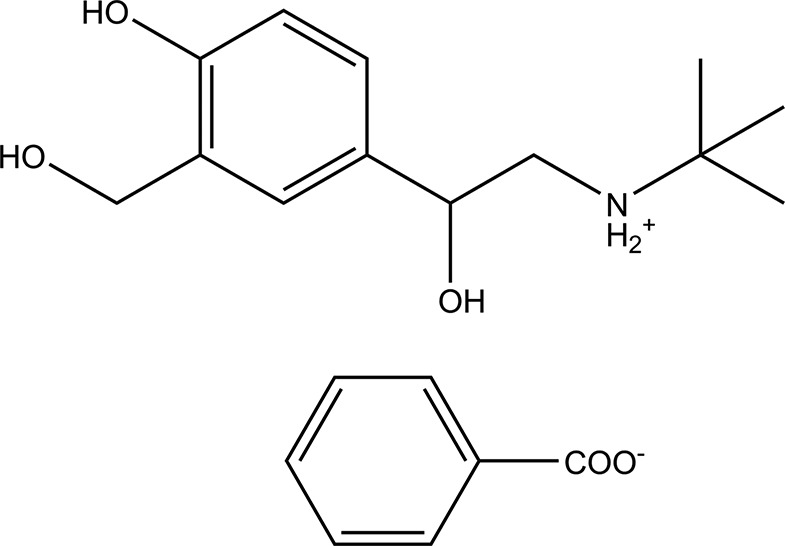


We selected various acids and combined them with salbutamol and then investigated the properties of new salt forms. Salbutamol benzoate was found to dissolve and crystallize in water, and it might show different *in vitro* solubility and dissolution properties. In this work, we report on the crystal structure determination of the title molecular salt salbutamol benzoate, C_13_H_22_NO_3_^+^·C_7_H_5_O_2_^−^.

## Structural commentary   

**Figure 1:**
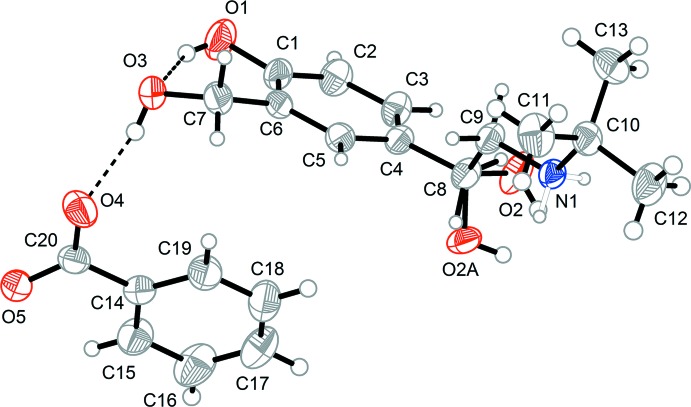
The structures of the mol­ecular components in the title compound. Displacement ellipsoids are drawn at the 50% probability level. The dashed line depicts the O—H⋯O hydrogen bond. Both disorder components of the OH group are shown.

The asymmetric unit of the title compound is shown in Fig. 1 ▸. The mol­ecule of salbutamol (**SAL**) accepts one proton at the N1 atom from the benzoic acid (**BA**) and thus forms a 1:1 salt, **SAL** ^+^ **BA** ^−^. The bond lengths of the carboxyl­ate group of the **BA** ^−^ anion, C20—O4 and C20—O5, are 1.2617 (15) and 1.2604 (15) Å, respectively. The slight difference may be caused by the role of O4 as an acceptor atom of the O3—H3⋯O4 hydrogen bond with one of the hy­droxy groups of **SAL^+^**. The **SAL^+^** cation also has an intra­molecular hydrogen bond between the two hy­droxy functions (O1—H1⋯O3), forming an *S*(6) ring motif (Fig. 1 ▸ and Table 1 ▸).

The **BA^−^** anion is not planar, indicated by the dihedral angle between the benzene ring and the carboxyl group of 11.30 (8)°. There is some disorder at the stereogenic centre (C8) of the **SAL^+^** cation, but the space group is centrosymmetric and the **SAL^+^** cation is racemic.

## Supra­molecular features   

**Figure 2:**
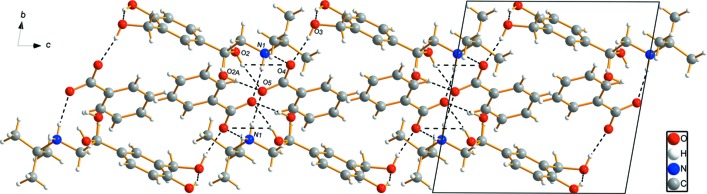
The crystal packing of the title compound, viewed perpendicular to the *bc* plane. N—H⋯O and O—H⋯O hydrogen bonds are shown as dashed lines (Table 1 ▸ gives the numerical details). Both disorder components of the OH group are shown.

The **SAL^+^** cation is connected to the **BA^−^** anion *via* a medium-strength O3—H3⋯O4 hydrogen bond (Table 1 ▸). In addition, N—H⋯O hydrogen bonds between **SAL^+^** and **BA^−^** are present, leading to an 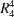
(12) graph-set motif *via* N1—H1*A*⋯O4^i^ and N1—H1*B*⋯O5^ii^ (for symmetry codes, see Table 1 ▸). Due to the disorder of the hy­droxy group at C8, there are some variable motifs, including 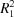
(4) motifs for the O2—H2⋯O4^i^ and O2*A*—H2*A*⋯O5^i^ inter­actions, respectively. The variety of N—H⋯·O and O—H⋯O hydrogen bonds leads to [001] chains (Figs. 2 ▸ and 3 ▸).

## Database survey   

Six structures containing salbutamol were found in a search of the Cambridge Structural Database (Version 5.38; Groom *et al.*, 2016 ▸). The structure of salbutamol was reported by Beale & Grainger (1972 ▸). Salbutamol sulfate was the first salt of salbutamol to be structurally determined some years later (Leger *et al.*, 1978 ▸). Recently, a new salbutamol sulfate polymorph crystallizing in a different space group (*C*2/*c*) was determined (Xie *et al.*, 2010 ▸). Paluch *et al.* (2011 ▸) investigated the co-crystal of a salbutamol hemiadipate salt with adipic acid and also the salbutamol hemisuccinate salt. Moreover, an oxaprozin–salbutamol salt was also reported (Aitipamula *et al.*, 2016 ▸).

## Synthesis and crystallization   

Salbutamol (0.479 g, 2 mmol) and benzoic acid (0.244 g, 2 mmol) were added to 10 ml methanol and stirred for 3 h. The solvent was then evaporated at room temperature to yield salbutamol benzoate. After recrystallization from water, pure crystals were again dissolved in ethanol and the solution filtered. The neat filtrate was evaporated slowly to give colourless block-like single crystals of salbutamol benzoate.

## Refinement   

Crystal data, data collection and structure refinement details are summarized in Table 2 ▸. The hy­droxy group at C8 is disordered over two sets of sites, with refined site occupancies of 0.738:0.262. H atoms were constrained to an ideal geometry, with C—H distances in the range 0.93–0.97 Å, and allowed to ride, with *U*_iso_(H) = 1.5*U*_eq_(C) for methyl H atoms and *U*_iso_(H) = 1.2*U*_eq_(C) for all other H atoms. The H atoms of the NH_2_ group and the hy­droxy group (except for O1—H1, which was refined freely) were also constrained to ideal values and allowed to ride in the refinement, with *U*_iso_(H) = 1.2*U*_eq_(N) and 1.5*U*_eq_(O).

## Supplementary Material

Crystal structure: contains datablock(s) I, New_Global_Publ_Block. DOI: 10.1107/S2056989017011513/wm5403sup1.cif

Structure factors: contains datablock(s) I. DOI: 10.1107/S2056989017011513/wm5403Isup2.hkl

Supporting information file. DOI: 10.1107/S2056989017011513/wm5403Isup3.cml

CCDC reference: 1482124

Additional supporting information:  crystallographic information; 3D view; checkCIF report

## Figures and Tables

**Figure 3:**
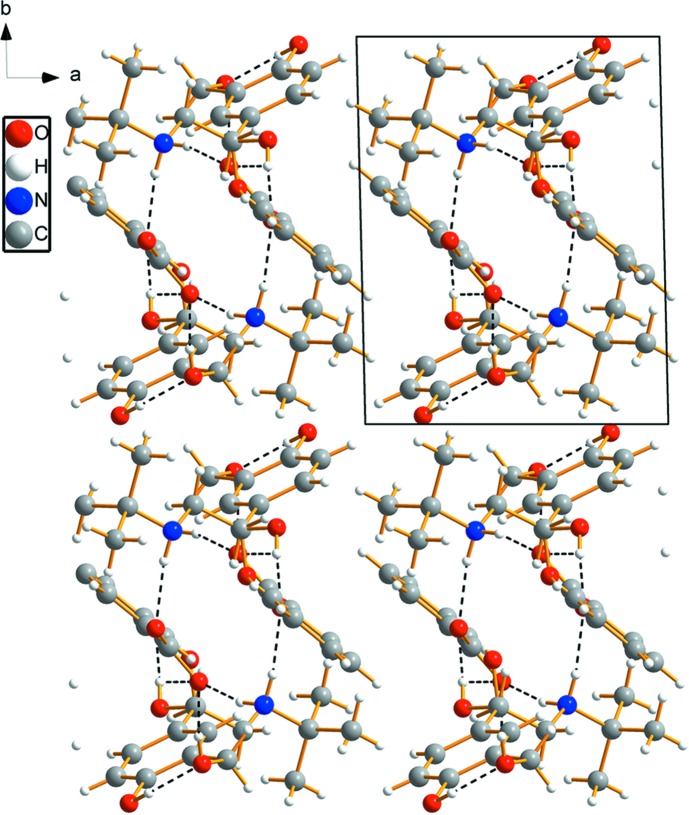
The crystal packing of the title compound, viewed perpendicular to the *ab* plane. N—H⋯O and O—H⋯O hydrogen bonds are shown as dashed lines (Table 1 ▸ gives the numerical details).

**Table 1:** Hydrogen-bond geometry (Å, °)

*D*—H⋯*A*	*D*—H	H⋯*A*	*D*⋯*A*	*D*—H⋯*A*
O2*A*—H2*A*⋯O5^i^	0.82	2.09	2.906 (4)	173
O2—H2⋯O4^i^	0.82	2.64	3.1066 (18)	118
O2—H2⋯O5^i^	0.82	1.89	2.7029 (16)	170
O3—H3⋯O4	0.82	1.83	2.6340 (15)	167
N1—H1*A*⋯O4^i^	0.89	1.99	2.8538 (14)	165
N1—H1*B*⋯O5^ii^	0.89	1.96	2.8452 (15)	171
O1—H1⋯O3	0.88 (2)	1.78 (2)	2.6015 (17)	154 (2)

Symmetry codes: (i) 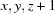; (ii) 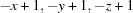.

**Table 2:** Experimental details

Crystal data	
Chemical formula	C_13_H_22_NO_3_^+^·C_7_H_5_O_2_^−^
*M*_r_	361.42
Crystal system, space group	Triclinic, *P* 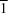
Temperature (K)	298
*a*, *b*, *c* (Å)	8.7525 (16), 10.691 (2), 11.220 (2)
α, β, γ (°)	79.953 (8), 69.969 (5), 87.796 (7)
*V* (Å^3^)	971.0 (3)
*Z*	2
Radiation type	Mo *K*α
μ (mm^−1^)	0.09
Crystal size (mm)	0.2 × 0.2 × 0.2

Data collection	
Diffractometer	Bruker APEXII CCD area detector
Absorption correction	Multi-scan (*SADABS*; Bruker, 2013 ▸)
*T*_min_, *T*_max_	0.702, 0.746
No. of measured, independent and observed [*I* > 2σ(*I*)] reflections	30220, 4451, 3646
*R*_int_	0.031
(sin θ/λ)_max_ (Å^−1^)	0.650

Refinement	
*R*[*F* ^2^ > 2σ(*F* ^2^)], *wR*(*F* ^2^), *S*	0.042, 0.112, 1.05
No. of reflections	4451
No. of parameters	255
H-atom treatment	H atoms treated by a mixture of independent and constrained refinement
Δρ_max_, Δρ_min_ (e Å^−3^)	0.20, −0.16

Computer programs: *SAINT* and *APEX2* (Bruker, 2013 ▸), *SHELXT* (Sheldrick, 2015*a* ▸), *SHELXL2014* (Sheldrick, 2015*b* ▸) and *OLEX2* (Dolomanov *et al.*, 2009 ▸).

## supplementary crystallographic information

### Crystal data

**Table:**

C_13_H_22_NO_3_^+^·C_7_H_5_O_2_^−^	*Z* = 2
*M**_r_* = 361.42	*F*(000) = 388
Triclinic, *P*1	*D*_x_ = 1.236 Mg m^−^^3^
*a* = 8.7525 (16) Å	Mo *K*α radiation, λ = 0.71073 Å
*b* = 10.691 (2) Å	Cell parameters from 9936 reflections
*c* = 11.220 (2) Å	θ = 2.6–27.5°
α = 79.953 (8)°	µ = 0.09 mm^−^^1^
β = 69.969 (5)°	*T* = 298 K
γ = 87.796 (7)°	Block, colourless
*V* = 971.0 (3) Å^3^	0.2 × 0.2 × 0.2 mm

### Data collection

**Table:**

Bruker APEXII CCD area detector diffractometer	3646 reflections with *I* > 2σ(*I*)
ω scans	*R*_int_ = 0.031
Absorption correction: multi-scan (*SADABS*; Bruker, 2013)	θ_max_ = 27.5°, θ_min_ = 2.6°
*T*_min_ = 0.702, *T*_max_ = 0.746	*h* = −11→11
30220 measured reflections	*k* = −13→13
4451 independent reflections	*l* = −14→14

### Refinement

**Table:**

Refinement on *F*^2^	Hydrogen site location: mixed
Least-squares matrix: full	H atoms treated by a mixture of independent and constrained refinement
*R*[*F*^2^ > 2σ(*F*^2^)] = 0.042	*w* = 1/[σ^2^(*F*_o_^2^) + (0.0446*P*)^2^ + 0.2722*P*] where *P* = (*F*_o_^2^ + 2*F*_c_^2^)/3
*wR*(*F*^2^) = 0.112	(Δ/σ)_max_ = 0.001
*S* = 1.05	Δρ_max_ = 0.20 e Å^−^^3^
4451 reflections	Δρ_min_ = −0.16 e Å^−^^3^
255 parameters	Extinction correction: SHELXL2014 (Sheldrick, 2015b), Fc^*^=kFc[1+0.001xFc^2^λ^3^/sin(2θ)]^-1/4^
0 restraints	Extinction coefficient: 0.057 (9)

### Special details

**Table:**

Geometry. All esds (except the esd in the dihedral angle between two l.s. planes) are estimated using the full covariance matrix. The cell esds are taken into account individually in the estimation of esds in distances, angles and torsion angles; correlations between esds in cell parameters are only used when they are defined by crystal symmetry. An approximate (isotropic) treatment of cell esds is used for estimating esds involving l.s. planes.

### Fractional atomic coordinates and isotropic or equivalent isotropic displacement parameters (Å^2^)

**Table:**

	*x*	*y*	*z*	*U*_iso_*/*U*_eq_	Occ. (<1)
O1	0.80860 (14)	0.97825 (11)	0.26965 (9)	0.0546 (3)	
O2A	0.5973 (5)	0.6081 (3)	0.8052 (3)	0.0434 (11)	0.262 (3)
H2A	0.6205	0.5820	0.8699	0.065*	0.262 (3)
O2	0.69965 (16)	0.73304 (15)	0.84687 (13)	0.0510 (5)	0.738 (3)
H2	0.6980	0.6657	0.8953	0.076*	0.738 (3)
O3	0.56450 (15)	0.87681 (9)	0.23563 (9)	0.0528 (3)	
H3	0.5737	0.8061	0.2153	0.079*	
N1	0.35552 (12)	0.72461 (9)	1.00143 (9)	0.0322 (2)	
H1A	0.4336	0.7173	1.0361	0.039*	
H1B	0.3251	0.6464	1.0005	0.039*	
C1	0.75065 (16)	0.91411 (12)	0.39463 (11)	0.0383 (3)	
C2	0.84945 (16)	0.90957 (14)	0.46798 (13)	0.0436 (3)	
H2B	0.9534	0.9462	0.4313	0.052*	
C3	0.79551 (15)	0.85081 (13)	0.59624 (12)	0.0391 (3)	
H3A	0.8623	0.8505	0.6453	0.047*	
C4	0.64238 (15)	0.79248 (11)	0.65167 (11)	0.0329 (3)	
C5	0.54602 (15)	0.79462 (11)	0.57543 (11)	0.0344 (3)	
H5	0.4446	0.7537	0.6109	0.041*	
C6	0.59620 (15)	0.85613 (11)	0.44730 (11)	0.0341 (3)	
C7	0.48166 (18)	0.86544 (13)	0.37203 (13)	0.0436 (3)	
H7A	0.4110	0.7904	0.4017	0.052*	
H7B	0.4138	0.9387	0.3889	0.052*	
C8	0.58179 (15)	0.72927 (12)	0.79268 (11)	0.0366 (3)	
H8A	0.5559	0.6399	0.7967	0.044*	0.738 (3)
H8B	0.6622	0.7569	0.8262	0.044*	0.262 (3)
C9	0.42675 (15)	0.79134 (12)	0.86485 (11)	0.0366 (3)	
H9A	0.4502	0.8795	0.8639	0.044*	
H9B	0.3481	0.7895	0.8217	0.044*	
C10	0.20953 (16)	0.78698 (13)	1.08980 (12)	0.0391 (3)	
C11	0.06894 (18)	0.79052 (17)	1.03926 (15)	0.0544 (4)	
H11A	0.1008	0.8395	0.9540	0.082*	
H11B	−0.0228	0.8287	1.0949	0.082*	
H11C	0.0399	0.7055	1.0370	0.082*	
C12	0.1660 (2)	0.70235 (17)	1.22185 (13)	0.0583 (4)	
H12A	0.0730	0.7355	1.2814	0.087*	
H12B	0.2564	0.7006	1.2520	0.087*	
H12C	0.1411	0.6177	1.2152	0.087*	
C13	0.2593 (2)	0.92015 (15)	1.09557 (15)	0.0532 (4)	
H13A	0.2799	0.9732	1.0131	0.080*	
H13B	0.3562	0.9161	1.1179	0.080*	
H13C	0.1733	0.9551	1.1594	0.080*	
O4	0.56936 (13)	0.66457 (10)	0.14546 (11)	0.0537 (3)	
O5	0.70749 (13)	0.52721 (9)	0.02270 (9)	0.0456 (3)	
C14	0.73821 (15)	0.51968 (11)	0.22649 (12)	0.0360 (3)	
C15	0.86636 (18)	0.43726 (15)	0.19863 (15)	0.0504 (4)	
H15	0.9083	0.4160	0.1168	0.061*	
C16	0.9327 (2)	0.38618 (18)	0.29166 (17)	0.0649 (5)	
H16	1.0185	0.3304	0.2721	0.078*	
C17	0.8726 (2)	0.41749 (17)	0.41258 (16)	0.0612 (4)	
H17	0.9173	0.3827	0.4749	0.073*	
C18	0.7469 (2)	0.50006 (17)	0.44133 (16)	0.0605 (4)	
H18	0.7069	0.5222	0.5228	0.073*	
C19	0.67932 (19)	0.55051 (14)	0.34893 (14)	0.0486 (3)	
H19	0.5932	0.6059	0.3693	0.058*	
H1	0.743 (2)	0.9521 (19)	0.2336 (19)	0.073*	
C20	0.66666 (16)	0.57426 (11)	0.12434 (13)	0.0369 (3)	

### Atomic displacement parameters (Å^2^)

**Table:**

	*U*^11^	*U*^22^	*U*^33^	*U*^12^	*U*^13^	*U*^23^
O1	0.0637 (7)	0.0627 (7)	0.0279 (5)	−0.0049 (5)	−0.0088 (4)	0.0047 (4)
O2A	0.058 (2)	0.0273 (17)	0.041 (2)	0.0088 (15)	−0.0153 (17)	0.0002 (14)
O2	0.0418 (7)	0.0683 (10)	0.0387 (7)	0.0011 (6)	−0.0185 (6)	0.0115 (6)
O3	0.0895 (8)	0.0417 (5)	0.0353 (5)	0.0097 (5)	−0.0304 (5)	−0.0105 (4)
N1	0.0357 (5)	0.0334 (5)	0.0254 (5)	0.0059 (4)	−0.0083 (4)	−0.0049 (4)
C1	0.0463 (7)	0.0374 (6)	0.0245 (6)	0.0059 (5)	−0.0052 (5)	−0.0037 (5)
C2	0.0368 (7)	0.0514 (8)	0.0353 (7)	−0.0009 (6)	−0.0057 (5)	−0.0018 (6)
C3	0.0357 (6)	0.0474 (7)	0.0326 (6)	0.0069 (5)	−0.0119 (5)	−0.0039 (5)
C4	0.0357 (6)	0.0320 (6)	0.0272 (6)	0.0091 (5)	−0.0069 (5)	−0.0044 (4)
C5	0.0369 (6)	0.0333 (6)	0.0307 (6)	0.0043 (5)	−0.0081 (5)	−0.0073 (5)
C6	0.0445 (7)	0.0304 (6)	0.0289 (6)	0.0096 (5)	−0.0126 (5)	−0.0106 (5)
C7	0.0578 (8)	0.0421 (7)	0.0376 (7)	0.0127 (6)	−0.0225 (6)	−0.0143 (5)
C8	0.0383 (6)	0.0385 (6)	0.0287 (6)	0.0090 (5)	−0.0089 (5)	−0.0015 (5)
C9	0.0410 (7)	0.0373 (6)	0.0263 (6)	0.0092 (5)	−0.0077 (5)	−0.0014 (5)
C10	0.0391 (7)	0.0445 (7)	0.0300 (6)	0.0090 (5)	−0.0058 (5)	−0.0109 (5)
C11	0.0384 (7)	0.0745 (11)	0.0497 (8)	0.0133 (7)	−0.0105 (6)	−0.0210 (7)
C12	0.0592 (9)	0.0708 (11)	0.0310 (7)	0.0076 (8)	−0.0005 (6)	−0.0044 (7)
C13	0.0613 (9)	0.0499 (8)	0.0497 (8)	0.0127 (7)	−0.0148 (7)	−0.0230 (7)
O4	0.0649 (7)	0.0524 (6)	0.0640 (7)	0.0283 (5)	−0.0411 (6)	−0.0295 (5)
O5	0.0633 (6)	0.0375 (5)	0.0384 (5)	0.0109 (4)	−0.0198 (4)	−0.0097 (4)
C14	0.0367 (6)	0.0315 (6)	0.0399 (7)	0.0014 (5)	−0.0135 (5)	−0.0053 (5)
C15	0.0493 (8)	0.0534 (8)	0.0453 (8)	0.0167 (7)	−0.0134 (6)	−0.0088 (6)
C16	0.0553 (9)	0.0714 (11)	0.0636 (10)	0.0264 (8)	−0.0223 (8)	−0.0014 (8)
C17	0.0624 (10)	0.0679 (11)	0.0547 (9)	0.0054 (8)	−0.0310 (8)	0.0078 (8)
C18	0.0762 (11)	0.0661 (10)	0.0424 (8)	0.0097 (8)	−0.0252 (8)	−0.0093 (7)
C19	0.0552 (8)	0.0480 (8)	0.0440 (8)	0.0145 (6)	−0.0179 (7)	−0.0125 (6)
C20	0.0404 (7)	0.0304 (6)	0.0428 (7)	0.0028 (5)	−0.0167 (5)	−0.0090 (5)

### Geometric parameters (Å, º)

**Table:**

O1—C1	1.3777 (15)	C9—H9A	0.9700
O1—H1	0.88 (2)	C9—H9B	0.9700
O2A—H2A	0.8200	C10—C11	1.518 (2)
O2A—C8	1.286 (3)	C10—C12	1.5267 (19)
O2—H2	0.8200	C10—C13	1.523 (2)
O2—C8	1.3700 (18)	C11—H11A	0.9600
O3—H3	0.8200	C11—H11B	0.9600
O3—C7	1.4368 (16)	C11—H11C	0.9600
N1—H1A	0.8900	C12—H12A	0.9600
N1—H1B	0.8900	C12—H12B	0.9600
N1—C9	1.4980 (15)	C12—H12C	0.9600
N1—C10	1.5354 (15)	C13—H13A	0.9600
C1—C2	1.3777 (19)	C13—H13B	0.9600
C1—C6	1.3964 (19)	C13—H13C	0.9600
C2—H2B	0.9300	O4—C20	1.2617 (15)
C2—C3	1.3894 (18)	O5—C20	1.2604 (15)
C3—H3A	0.9300	C14—C15	1.3827 (19)
C3—C4	1.3892 (18)	C14—C19	1.3862 (19)
C4—C5	1.3891 (17)	C14—C20	1.5088 (18)
C4—C8	1.5234 (16)	C15—H15	0.9300
C5—H5	0.9300	C15—C16	1.385 (2)
C5—C6	1.3971 (17)	C16—H16	0.9300
C6—C7	1.5070 (18)	C16—C17	1.373 (2)
C7—H7A	0.9700	C17—H17	0.9300
C7—H7B	0.9700	C17—C18	1.370 (2)
C8—H8A	0.9800	C18—H18	0.9300
C8—H8B	0.9800	C18—C19	1.384 (2)
C8—C9	1.5175 (17)	C19—H19	0.9300
			
C1—O1—H1	103.7 (13)	C8—C9—H9A	109.4
C8—O2A—H2A	109.5	C8—C9—H9B	109.4
C8—O2—H2	109.5	H9A—C9—H9B	108.0
C7—O3—H3	109.5	C11—C10—N1	109.36 (10)
H1A—N1—H1B	107.3	C11—C10—C12	110.57 (13)
C9—N1—H1A	108.1	C11—C10—C13	111.26 (12)
C9—N1—H1B	108.1	C12—C10—N1	105.47 (11)
C9—N1—C10	116.66 (9)	C13—C10—N1	109.25 (11)
C10—N1—H1A	108.1	C13—C10—C12	110.75 (12)
C10—N1—H1B	108.1	C10—C11—H11A	109.5
O1—C1—C2	117.93 (12)	C10—C11—H11B	109.5
O1—C1—C6	121.77 (12)	C10—C11—H11C	109.5
C2—C1—C6	120.30 (11)	H11A—C11—H11B	109.5
C1—C2—H2B	119.6	H11A—C11—H11C	109.5
C1—C2—C3	120.70 (12)	H11B—C11—H11C	109.5
C3—C2—H2B	119.6	C10—C12—H12A	109.5
C2—C3—H3A	119.8	C10—C12—H12B	109.5
C4—C3—C2	120.42 (12)	C10—C12—H12C	109.5
C4—C3—H3A	119.8	H12A—C12—H12B	109.5
C3—C4—C8	120.65 (11)	H12A—C12—H12C	109.5
C5—C4—C3	118.20 (11)	H12B—C12—H12C	109.5
C5—C4—C8	121.15 (11)	C10—C13—H13A	109.5
C4—C5—H5	118.9	C10—C13—H13B	109.5
C4—C5—C6	122.27 (12)	C10—C13—H13C	109.5
C6—C5—H5	118.9	H13A—C13—H13B	109.5
C1—C6—C5	118.06 (11)	H13A—C13—H13C	109.5
C1—C6—C7	121.60 (11)	H13B—C13—H13C	109.5
C5—C6—C7	120.24 (12)	C15—C14—C19	118.43 (13)
O3—C7—C6	113.03 (12)	C15—C14—C20	119.93 (12)
O3—C7—H7A	109.0	C19—C14—C20	121.64 (12)
O3—C7—H7B	109.0	C14—C15—H15	119.8
C6—C7—H7A	109.0	C14—C15—C16	120.49 (14)
C6—C7—H7B	109.0	C16—C15—H15	119.8
H7A—C7—H7B	107.8	C15—C16—H16	119.8
O2A—C8—C4	111.63 (19)	C17—C16—C15	120.32 (15)
O2A—C8—H8B	103.5	C17—C16—H16	119.8
O2A—C8—C9	122.7 (2)	C16—C17—H17	120.0
O2—C8—C4	111.00 (11)	C18—C17—C16	119.92 (15)
O2—C8—H8A	108.0	C18—C17—H17	120.0
O2—C8—C9	111.94 (12)	C17—C18—H18	120.0
C4—C8—H8A	108.0	C17—C18—C19	119.93 (15)
C4—C8—H8B	103.5	C19—C18—H18	120.0
C9—C8—C4	109.70 (10)	C14—C19—H19	119.5
C9—C8—H8A	108.0	C18—C19—C14	120.90 (14)
C9—C8—H8B	103.5	C18—C19—H19	119.5
N1—C9—C8	111.32 (9)	O4—C20—C14	118.13 (11)
N1—C9—H9A	109.4	O5—C20—O4	123.67 (12)
N1—C9—H9B	109.4	O5—C20—C14	118.20 (11)
			
O1—C1—C2—C3	177.38 (12)	C5—C4—C8—C9	−58.81 (15)
O1—C1—C6—C5	−179.09 (11)	C5—C6—C7—O3	−153.65 (11)
O1—C1—C6—C7	−2.70 (18)	C6—C1—C2—C3	−2.0 (2)
O2A—C8—C9—N1	41.1 (3)	C8—C4—C5—C6	177.49 (11)
O2—C8—C9—N1	−61.14 (15)	C9—N1—C10—C11	62.74 (15)
C1—C2—C3—C4	1.8 (2)	C9—N1—C10—C12	−178.33 (11)
C1—C6—C7—O3	30.04 (16)	C9—N1—C10—C13	−59.25 (14)
C2—C1—C6—C5	0.23 (18)	C10—N1—C9—C8	173.01 (11)
C2—C1—C6—C7	176.62 (12)	C14—C15—C16—C17	−0.4 (3)
C2—C3—C4—C5	0.10 (19)	C15—C14—C19—C18	0.0 (2)
C2—C3—C4—C8	−179.27 (12)	C15—C14—C20—O4	−168.35 (13)
C3—C4—C5—C6	−1.87 (18)	C15—C14—C20—O5	11.59 (19)
C3—C4—C8—O2A	−100.1 (2)	C15—C16—C17—C18	−0.3 (3)
C3—C4—C8—O2	−3.70 (17)	C16—C17—C18—C19	0.8 (3)
C3—C4—C8—C9	120.54 (13)	C17—C18—C19—C14	−0.6 (3)
C4—C5—C6—C1	1.71 (17)	C19—C14—C15—C16	0.6 (2)
C4—C5—C6—C7	−174.73 (11)	C19—C14—C20—O4	11.3 (2)
C4—C8—C9—N1	175.17 (10)	C19—C14—C20—O5	−168.78 (13)
C5—C4—C8—O2A	80.6 (2)	C20—C14—C15—C16	−179.78 (14)
C5—C4—C8—O2	176.96 (12)	C20—C14—C19—C18	−179.68 (14)

### Hydrogen-bond geometry (Å, º)

**Table:**

*D*—H···*A*	*D*—H	H···*A*	*D*···*A*	*D*—H···*A*
O2*A*—H2*A*···O5^i^	0.82	2.09	2.906 (4)	173
O2—H2···O4^i^	0.82	2.64	3.1066 (18)	118
O2—H2···O5^i^	0.82	1.89	2.7029 (16)	170
O3—H3···O4	0.82	1.83	2.6340 (15)	167
N1—H1*A*···O4^i^	0.89	1.99	2.8538 (14)	165
N1—H1*B*···O5^ii^	0.89	1.96	2.8452 (15)	171
O1—H1···O3	0.88 (2)	1.78 (2)	2.6015 (17)	154 (2)

Symmetry codes: (i) *x*, *y*, *z*+1; (ii) −*x*+1, −*y*+1, −*z*+1.
